# Spatiotemporal transmission dynamics of co-circulating dengue, Zika, and chikungunya viruses in Fortaleza, Brazil: 2011–2017

**DOI:** 10.1371/journal.pntd.0008760

**Published:** 2020-10-26

**Authors:** Lilit Kazazian, Antonio S. Lima Neto, Geziel S. Sousa, Osmar José do Nascimento, Marcia C. Castro

**Affiliations:** 1 Department of Global Health and Population, Harvard T.H. Chan School of Public Health, Boston, Massachusetts, United States of America; 2 Health Surveillance Department, Fortaleza Municipal Health Secretariat (SMS-Fortaleza), Joaquim Távora, Fortaleza, Ceará, Brazil; 3 Health Sciences Center, University of Fortaleza (UNIFOR), Edson Queiroz, Fortaleza, Ceará, Brazil; University of California, Berkeley, UNITED STATES

## Abstract

The mosquito-borne viruses dengue (DENV), Zika (ZIKV), and chikungunya (CHIKV), now co-endemic in the Americas, pose growing threats to health worldwide. However, it remains unclear whether there exist interactions between these viruses that could shape their epidemiology. This study advances knowledge by assessing the transmission dynamics of co-circulating DENV, ZIKV, and CHIKV in the city of Fortaleza, Brazil. Spatiotemporal transmission dynamics of DENV, ZIKV, and CHIKV were analyzed using georeferenced data on over 210,000 reported cases from 2011 to 2017 in Fortaleza, Brazil. Local spatial clustering tests and space-time scan statistics were used to compare transmission dynamics across all years. The transmission of co-circulating viruses in 2016 and 2017 was evaluated at fine spatial and temporal scales using a measure of spatiotemporal dependence, the τ-statistic. Results revealed differences in the diffusion of CHIKV compared to previous DENV epidemics and spatially distinct transmission of DENV/ZIKV and CHIKV during the period of their co-circulation. Significant spatial clustering of viruses of the same type was observed within 14-day time intervals at distances of up to 6.8 km (*p*<0.05). These results suggest that arbovirus risk is not uniformly distributed within cities during co-circulation. Findings may guide outbreak preparedness and response efforts by highlighting the clustered nature of transmission of co-circulating arboviruses at the neighborhood level. The potential for competitive interactions between the arboviruses should be further investigated.

## Author summary

Most of the global population is at risk of infection with dengue, Zika, and chikungunya viruses, all transmitted by the *Aedes* mosquitoes. Although these viruses now circulate together in the Americas, it is not known how, if at all, they might interact inside co-infected vectors or hosts. Such interactions could serve as important determinants of transmission in the context of co-circulation. We used seven years of surveillance data from Fortaleza, the fifth largest city in Brazil, to investigate the potential effects of co-circulation on the transmission dynamics of the three *Aedes*-borne viruses. We found that, during years with co-circulation, different families of viruses (dengue/Zika and chikungunya) clustered in distinct areas of the city. This spatial clustering of viruses of the same type was maintained even within narrow time intervals. Transmission patterns in epidemic years with co-circulation deviated from patterns in previous epidemic years. Our findings suggest that co-circulation may alter the transmission dynamics of these mosquito-borne viruses. Further research is needed to confirm any competitive interactions between the viruses and to establish evidence-based public health recommendations for concurrent outbreaks.

## Introduction

Mosquito-borne viruses pose a mounting challenge in the context of climate change, rapid urbanization, and inadequate access to infrastructure [1,2]. Dengue (DENV), Zika (ZIKV), and chikungunya (CHIKV) viruses, all transmitted by the *Aedes* mosquitoes, are now endemic to much of the Americas [1]. Nearly four billion people worldwide are estimated to be at risk of a DENV infection, and the share at risk of ZIKV and CHIKV is expected to rise [1,3]. In their acute phases, these infections share similar clinical presentations of fever, rash, and joint pain. More severe complications include dengue hemorrhagic fever, congenital Zika syndrome (CZS), and post-chikungunya chronic inflammatory joint disease [1]. The co-circulation of these viruses increases the likelihood of consecutive and simultaneous infections, the clinical and epidemiological ramifications of which remain poorly understood [4,5]. Improving the preparedness and response to arbovirus outbreaks, as well as treatment management efforts, requires a better understanding of transmission dynamics in the context of co-circulation, including how these viruses interact and spread [6,7].

The *Ae*. *aegypti* mosquito is responsible for most of the transmission of DENV, ZIKV, and CHIKV in tropical urban settings worldwide [8–10]. Densely populated cities and peri-urban areas serve as excellent habitats for this vector due to its preference for human blood meals and human-made breeding habitats, including varied water containers and tires [11,12]. The cyclical and seasonal transmission patterns of DENV, CHIKV, and ZIKV depend upon complex interactions between the virus, its mosquito and human hosts, and the environment [13]. The co-circulation of multiple arboviruses may introduce novel between-virus interactions, further complicating transmission dynamics. Transmission dynamics of co-circulating arboviruses may be mediated by the intrinsic properties of each virus and vector (i.e., differential replication rates or vector competence), viral interference in co-infected hosts (i.e., competitive suppression), or immunological effects (i.e., cross-protection or antibody-dependent enhancement) [14–16]. If no interactions are present, each virus should transmit independently of the others in space and time. In cities where these arboviruses co-circulate, interactions between them might only be visible at the neighborhood level [17], which is the relevant resolution for guiding local vector control efforts [13].

The relationships between these viruses remain elusive. Although a single *Ae*. *aegypti* mosquito has the capacity to transmit all three viruses in one bite [18], some studies have demonstrated competitive suppression between CHIKV, ZIKV, and/or DENV in co-infected mosquitoes [19,20], while others have found no significant impact of co-infection on infection or transmission rates [18,21,22]. Even without competitive suppression, differences in infection and transmission capacity between viruses in infected mosquitoes could shape transmission dynamics on an ecological scale. However, laboratory studies of vector competence yield highly variable results due to sensitivity to viral dose, virus genetics, mosquito species, and environmental conditions [23–25]. Finally, there has been increasing interest in the topic of immunological interactions between the flaviviruses DENV and ZIKV [15,26]. Some cross-neutralization by immune sera or T cells has been demonstrated *in vitro*, with limited epidemiological work to support this finding [27–31]. In contrast, extensive antibody-dependent enhancement has been shown *in vitro*, but attempts to extend this result to *in vivo* models of infection have produced mixed results [32–35].

In the Americas, transmission of DENV began as early as the 1600s, while CHIKV and ZIKV were introduced to the western hemisphere only in the 21^st^ century [1,36]. After being eliminated during the 1950s, DENV reemerged in Brazil in the 1980s [37] and has been hyperendemic since then, leading to as many as 1.69 million annual cases [38]. CHIKV was most likely introduced to Brazil in 2013 [39,40], with 463,619 probable cases reported in 2016 and 2017 [38]. Similarly, genetic analyses place the introduction of ZIKV in Brazil in 2013 [41,42], although the first cases and associations between ZIKV and Guillain-Barré syndrome and microcephaly in babies were reported in 2015 [43]. Brazil reported 215,319 probable Zika cases in 2016 when the mandatory notification for Zika was instituted nationwide [38], but the incidence has since declined [44]. Of the 1,950 confirmed CZS cases from 2015 to 2016, most occurred in northeast Brazil [45]. These official case counts are likely to underestimate true disease burden due to limitations of the surveillance system and diagnostic challenges, including the poor sensitivity and specificity of existing clinical case definitions [46,47].

DENV, ZIKV, and CHIKV co-circulate in Fortaleza, the capital city of the state of Ceará, located in the Northeast region of Brazil. Approximately 40% of the city’s inhabitants reside in precarious settlements, where poor housing, irregular waste collection, and deficient water and sanitation infrastructure contribute to the spread of vector-borne diseases [48]. DENV-1 was reintroduced to Fortaleza in 1986, and all four serotypes have since caused outbreaks [48]. The city has recorded five years of epidemic DENV transmission (over 20,000 reported cases) in between years of interepidemic transmission [48]. The first reports of ZIKV and CZS in Fortaleza occurred in 2015, but case detection efforts were hampered by the similarity of DENV and ZIKV clinical presentations, and by the absence of a sensitive serological test [46–48]. Fortaleza experienced its first two epidemic waves of CHIKV in 2016 and 2017. The second wave was much more pronounced, recording the highest number of reported cases of CHIKV in Brazil (57,226 out of 151,966) and of CHIKV-related deaths (128 out of 173) [44,48].

This paper aims to advance current knowledge of the spatiotemporal transmission dynamics of co-circulating arboviruses in a large urban setting. We use clinically- or laboratory-confirmed dengue, chikungunya, and Zika cases over a seven-year period (2011–2017) in the city of Fortaleza. We chose Fortaleza for three main reasons: (i) during the study period, the city observed the reintroduction of DENV-1 in 2011 (after 10 years of no circulation), the introduction of DENV-4 in 2012, and the introduction of ZIKV and CHIKV, (ii) the city had the largest number of chikungunya cases in Brazil, and (iii) data for Fortaleza were obtained at the finest spatial and temporal scales. Surveillance data from 2011 to 2017 allowed comparison of transmission dynamics before and after multiple viruses began co-circulating. Findings may help target surveillance and outbreak response activities in Fortaleza and other settings experiencing the triple burden of DENV, CHIKV, and ZIKV.

## Materials and methods

### Ethics statement

All data used in this analysis were anonymized. Study approval was obtained from the Institutional Review Board (IRB) of the Harvard T.H. Chan School of Public Health, Protocol #IRB18-0810.

### Study area

Fortaleza is the capital of the state of Ceará in the Northeast region of Brazil. It is the fifth most populous city in the country, with an estimated population of 2.6 million in 2018 [49]. Administratively, Fortaleza is divided into six *regionais* (administrative districts) and 119 *bairros* (administrative sub-districts, or city neighborhoods); *bairros* were chosen as the spatial unit of analysis in this study. A *bairro* name map and key are included in the supplementary material (S1 Fig and S1 Table).

The DENV epidemic in 2011 was caused by the reintroduction of a serotype that had been absent for 10 years (DENV-1), while the epidemic in 2012 was brought on by the introduction of a novel serotype (DENV-4). DENV-1 returned in 2014 and remained as the dominant DENV serotype until the end of the study period. Cases of ZIKV and CZS were first reported in 2015 and continued widely into 2016. The first autochthonous case of CHIKV was detected in December 2015, and two epidemic waves of CHIKV ensued in 2016 and 2017. CHIKV, DENV-1, and ZIKV co-circulated to some extent in 2016 and 2017.

### Data sources

Cases of dengue, chikungunya and Zika were collected by Fortaleza’s Epidemiological Surveillance Department (CEVEPI), which developed an application called *Sistema de Monitoramento Diário de Agravos* (SIMDA) aimed at facilitating surveillance and programmatic response [48]. Physicians are mandated to report all suspected arbovirus cases to Brazil’s Notifiable Diseases Information System (*Sistema de Informação de Agravos de Notificação*, SINAN) [50]. Within 60 days of reporting, suspected cases from SINAN are confirmed and added to SIMDA by CEVEPI based on laboratory tests or clinical/epidemiological criteria [48]. SIMDA contains any available demographic, clinical, and laboratory data linked to each confirmed arbovirus case. These cases are georeferenced to the residence of the individual and detailed by day and month based on the date of onset of symptoms. In cases where the individual’s address is ambiguous, cases are mapped to the centroid of the *bairro*. In 2016 and 2017, 12,509 cases (11.5%) were mapped to centroids, and these points were spatially randomly redistributed within *bairros* for analyses requiring point data.

This study included all clinically- or laboratory-confirmed dengue, Zika, and chikungunya cases from SIMDA with dates of onset of symptoms between January 1, 2011, and November 31, 2017. The introduction of ZIKV in 2015 reduced the diagnostic accuracy for DENV, as both are flaviviruses with similar clinical presentations and cross-reactive antibodies [1,46]. Therefore, we considered dengue cases from 2015 to 2017 to be indistinguishable from Zika cases and combined the two in the analyses. We used any available laboratory data linked to each case to calculate the positivity of cases from SIMDA, defined as the number of laboratory-confirmed cases as a proportion of total clinically- and laboratory-confirmed cases. Because it is not feasible from a programmatic perspective to run laboratory tests for all cases, Brazil’s Ministry of Health has recommended maintaining a case positivity of at least 10% during years with epidemic DENV transmission [51]. The guidelines were modified in 2017 after the introduction of CHIKV to recommend testing of only severe cases and new cases in *bairros* that had not previously reported autochthonous transmission [52]. In periods of non-epidemic DENV transmission, the Ministry of Health recommends testing of all suspected dengue cases from the sixth day after symptom onset [52].

Additional data from Fortaleza’s central laboratory, *Gerenciador de Ambiente Laboratorial* (GAL), were used to reclassify clinically-confirmed dengue cases based on suspected misdiagnosis between dengue and chikungunya cases in 2016 and 2017. GAL captures all positive, negative, and inconclusive IgM Enzyme-Linked Immunosorbent Assays (ELISAs) from tested individuals who may or may not be included as confirmed cases in SIMDA. Blood samples for testing by DENV- or CHIKV-IgM ELISA were typically collected from suspected cases when they sought care at primary healthcare units or emergency care services during the acute phase of an illness. No ZIKV-IgM ELISAs were performed during the study period. The positivity of laboratory tests from GAL (suspected case positivity) was defined as the number of positive DENV- or CHIKV-IgM ELISAs as a proportion of total DENV- or CHIKV-IgM ELISAs performed on suspected cases. The GAL laboratory test positivity was expected to be substantially higher than the SIMDA case positivity during epidemic years when laboratory testing was discouraged past the recommended 10% case positivity threshold.

Total population by *bairro* was obtained from the 2010 Demographic Census and used as denominators to calculate incidence rates [53]. We also created a binary variable to distinguish between peak and low transmission seasons, where peak seasons included months with more than 10% of total annual cases per year, and low seasons included all other months. Data preparation was conducted in Stata v.15 (Stata Corp., College Station, TX, USA), and mapping and data visualization were performed in ArcMap v.10.3 (ESRI, Redlands, CA, USA) and R v.3.5.1.

### Reclassification of case data

**Fig 1:**
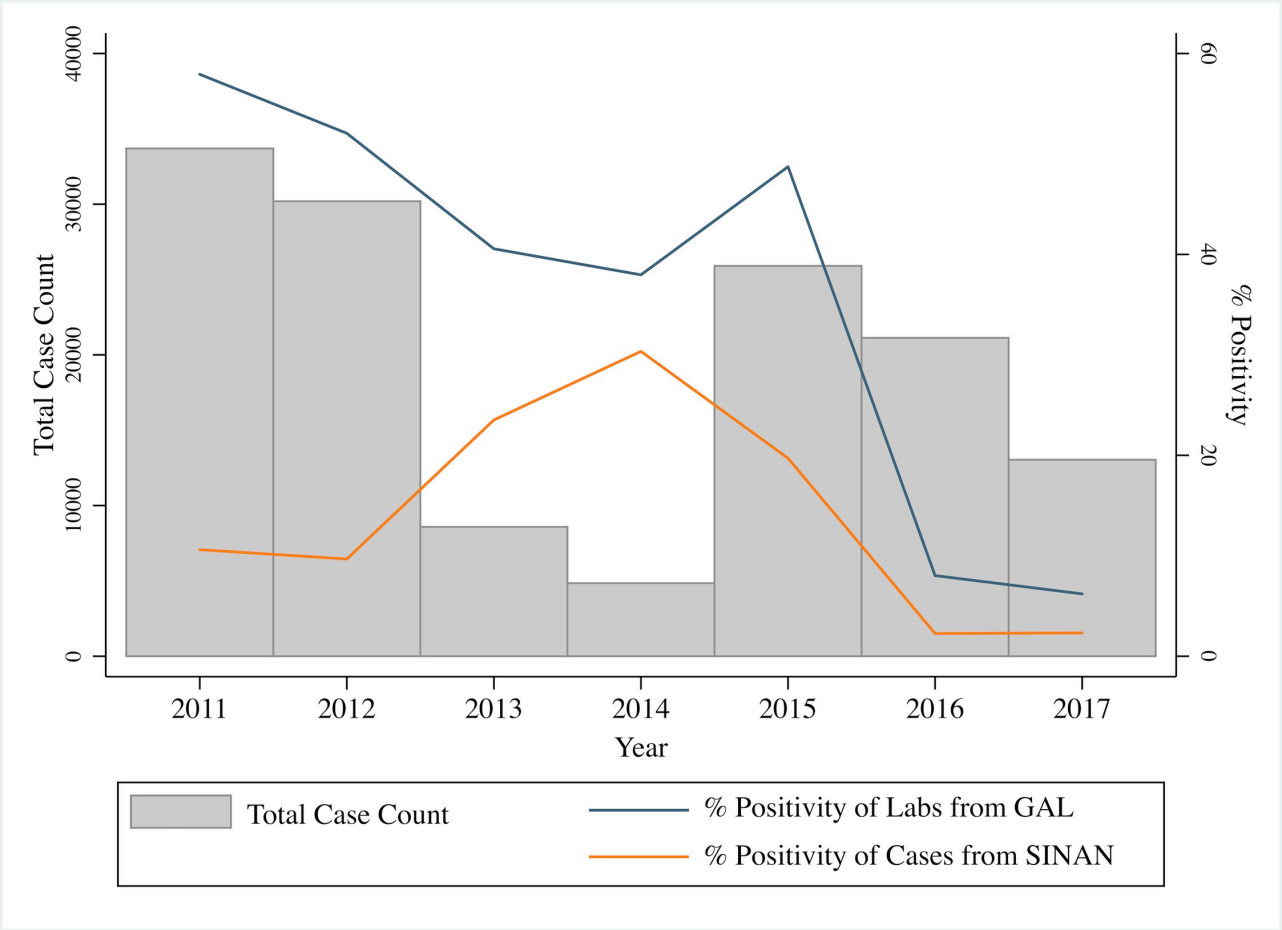
Case count and positivity of confirmed and suspected dengue cases, 2011–2017. Bars describe the total annual case count of confirmed dengue cases from SIMDA (left Y-axis). The right Y-axis indicates the DENV-IgM positivity of annual laboratory tests from GAL (suspected cases, blue line), and of annual dengue cases from SIMDA (orange line).

Following the introduction of CHIKV in 2016, the positivity of DENV-IgM ELISAs from GAL (suspected case positivity) dropped to 8.10% in 2016 and 6.16% in 2017, in sharp contrast with the positivity range of 37.96–58.01% observed from 2011–2015 (Fig 1). At the same time, the number of confirmed dengue cases in SIMDA remained at or above levels observed during interepidemic years (2013 and 2014). As a result, the SIMDA case positivity fell to nearly zero, despite the performance of thousands of serological tests on suspected dengue cases. Positivity of CHIKV-IgM tests from GAL remained over 50% during the same period (53.17% in 2016 and 75.01% in 2017).

**Fig 2:**
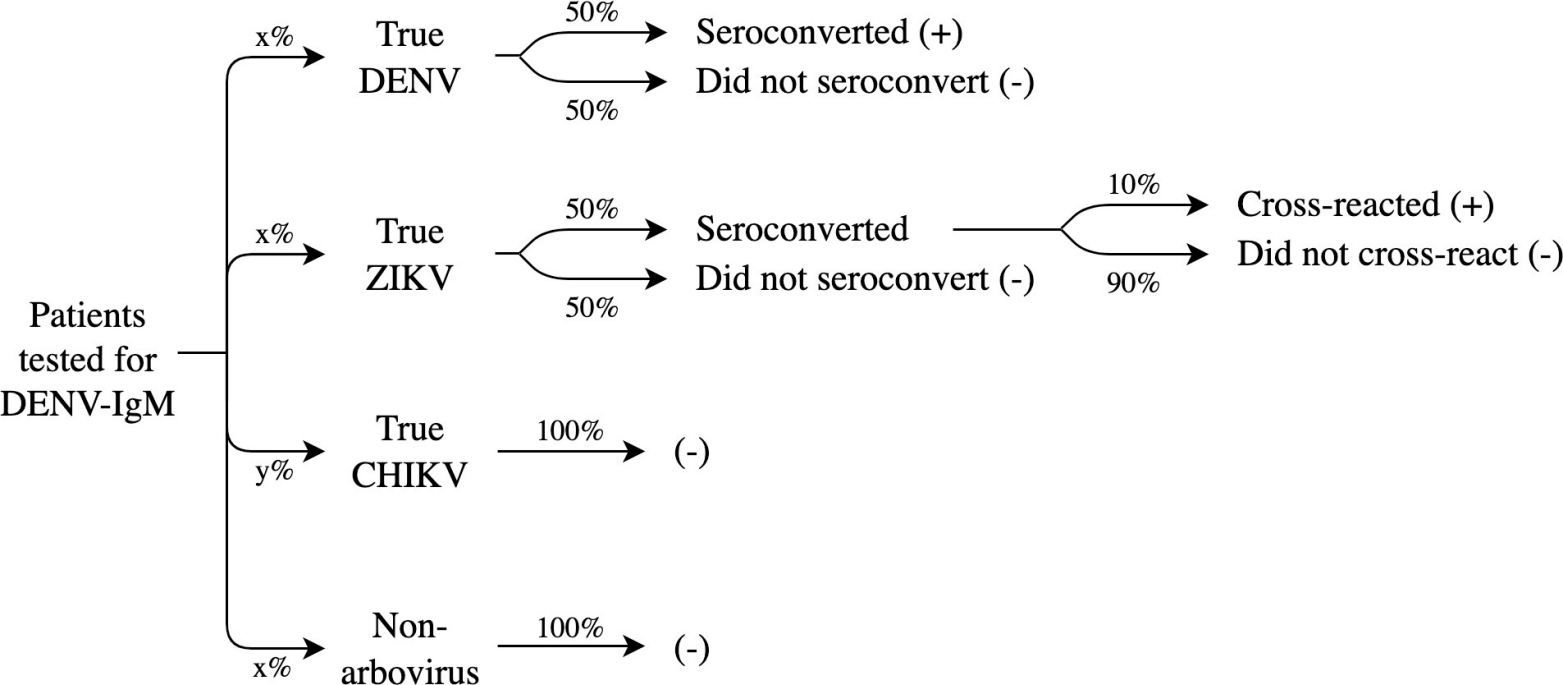
Algorithm for reclassifying dengue cases as chikungunya cases, 2016–2017. Patients tested for DENV-IgM in GAL were considered suspected dengue cases. Positive and negative DENV-IgM test results are indicated by (+) and (-), respectively.

Misclassification of arbovirus cases is expected when using symptoms-based diagnostic criteria. However, the pronounced decline in dengue suspected case positivity suggests higher-than-expected rates of misdiagnosis of true chikungunya cases as dengue in 2016 and 2017. Therefore, we randomly reclassified a proportion of clinically-confirmed dengue cases as chikungunya, based on an algorithm that relies on assumptions regarding both the underlying distribution of suspected cases and the probability of dengue/Zika cases testing positive on DENV-IgM ELISA (Fig 2). Because dengue and Zika cases were considered indistinguishable, cases were not reclassified if assumed to be Zika cases. The absence of ZIKV-IgM ELISAs during the study period prevented us from evaluating potential misclassification of Zika cases. The algorithm assumed that the probability of misdiagnosis was equal across *bairros*. Thus, the reclassification was applied globally across Fortaleza, reducing the magnitude of the DENV/ZIKV epidemic and increasing the magnitude of the CHIKV epidemic.

The algorithm for reclassifying dengue cases as chikungunya cases considered the following assumptions about patients tested for DENV-IgM in GAL, where unknown probabilities were assumed to be equal in the absence of empirical data to suggest otherwise:

1. In 2016 and 2017, patients tested for DENV-IgM had an equal probability (x%) of being a true dengue case, a true Zika case, or a non-arbovirus case. The remaining patients tested for DENV-IgM were true chikungunya cases (y%).
2. True dengue and Zika cases had a 50% probability of being tested prior to IgM antibody seroconversion.
3. 10% of Zika cases who seroconverted also presented as false positives in the DENV-IgM ELISA, consistent with known cross-reactivity rates from ZIKV convalescent serum samples (46).

Under these assumptions, a minimum positivity of 25% would be expected in the absence of co-circulating ZIKV and CHIKV, with negative test results attributable to non-arboviruses (50%) and DENV before seroconversion (25%). Prior to 2016, the positivity of DENV-IgM ELISAs had not fallen below this level.

Patients tested for DENV-IgM in GAL were considered representative of all suspected dengue cases. Thus, we estimated the proportion of true chikungunya cases among suspected dengue cases in SIMDA by solving for the proportion of true chikungunya cases among those tested for DENV-IgM in GAL (y%). In 2016, 50.1% of all suspected dengue cases were confirmed as dengue in SIMDA; in 2017, 36.8% of suspected dengue cases were confirmed (48). We applied these proportions to estimate the number of true chikungunya cases who were falsely confirmed as dengue cases and should be reclassified as chikungunya cases:

$$\begin{array}{l} \mathrm{Proportion}\;\mathrm{true}\;\mathrm{chikungunya}\;\mathrm{cases}\;\mathrm{among}\;\mathrm{confirmed}\;\mathrm{dengue}\;\mathrm{cases}=\\ \;\frac{y}{100}*\;\mathrm{proportion}\;\mathrm{confirmed}\;\mathrm{dengue}\;\mathrm{cases}\;\mathrm{among}\;\mathrm{suspected}\;\mathrm{dengue}\;\mathrm{cases} \end{array}$$

where:

$$\frac{y}{100}=1-\frac{3x}{100}$$

$$\frac{x}{100}=\frac{\mathrm{Observed}\;\mathrm{positivity}}{\left(0.50+0.50\;*\;0.10\right)}$$

Here, *Observed positivity* refers to the suspected case positivity of DENV-IgM laboratory tests from GAL each year, and *proportion confirmed dengue cases among suspected dengue cases* refers to the yearly proportions of suspected dengue cases confirmed as dengue in SIMDA. The proportion of true chikungunya cases among confirmed dengue cases was estimated at 28.0% in 2016 and 24.4% in 2017. We randomly selected these proportions of clinically-confirmed dengue cases each year to reclassify as chikungunya cases; numbers and proportions by *bairro* are presented in S2 Table. Remaining dengue cases were then combined with confirmed Zika cases to comprise the DENV/ZIKV category.

### Spatiotemporal analysis

To assess whether DENV, DENV/ZIKV, and CHIKV incidence rates were clustered in Fortaleza in each year of the study period, the Getis-Ord G* local test of spatial autocorrelation was applied [54]. A first-order queen’s contiguity matrix was selected to define the *bairros* neighborhood structure. Results were adjusted for multiple testing using the False Discovery Rate [55]. Calculations were done in ArcMap v.10.3 (ESRI, Redlands, CA, USA).

To investigate clustering patterns across both space and time, the Kulldorff’s space-time scan statistic was used [56], considering a discrete Poisson model. The spatial scanning area considered any *bairros* within a circle with a maximum radius of 2.5 km. To define this threshold, we assessed spatial autocorrelation in 500 m increments using Global Moran’s I for DENV, DENV/ZIKV, and CHIKV incidence rates each year and chose the median distance at which the z-scores reached their first peak. The maximum temporal scanning period was set at 14 days, which is the approximate duration of the *Ae*. *aegypti* life extrinsic incubation period [57]. Calculations were done using SaTScan v.9.6, and visualization of the results was done in ArcMap v.10.3 (ESRI, Redlands, CA, USA).

The previous two tests reveal patterns of disease clustering but do not provide an interpretation of disease risk at fine spatial and temporal scales during co-circulation. To measure the spatiotemporal dependence in transmission risk of DENV/ZIKV and CHIKV when they co-circulated in 2016 and 2017, we calculated the τ-statistic [17]. We determined the spatial dependence of cases of the same virus within 14-day intervals as given by τ(*d*_*1*_, *d*_*2*_)–the probability of two cases occurring within 14 days of one another and within a distance range d_1_ to d_2_ being the same virus, compared with the probability of any two cases within that time interval being the same virus [17]:

$$\tau (d_{1},d_{2})=\frac{\mathrm{Pr}\left(z_{i}=z_{j}\right|j\in \Omega_{i}(d_{1},d_{2}))}{\mathrm{Pr}\left(z_{i}=z_{j}\right|j\in \Omega_{i}(\cdot ))}$$

where Ω*i(d*_*1*_, *d*_*2*_*)* is the set of cases occurring during the same 14-day period and within distances *d*_*1*_ and *d*_*2*_ of case *i*, Ω*i*(·) is the set of all cases occurring in the same 14-day period, and *z*_*i*_ is the virus type of case *i*.

As in the space-time cluster analysis, a 14-day interval was chosen to reflect the approximate duration of the *Ae*. *aegypti* life cycle [57]. Distance ranges were calculated in 100 m increments from 0 m to 10 km, encompassing most of the area of Fortaleza. The spatiotemporal distribution of cases within a given 14-day period is present in both the numerator and denominator of the τ(*d*_*1*_, *d*_*2*_) formula. Thus, this model inherently controls for any variables which could account for spatial or temporal clustering, such as differential case reporting rates or population densities across *bairros* [17]. Calculations were done using the IDSpatialStats package in R v.3.5.1.

## Results

**Fig 3:**
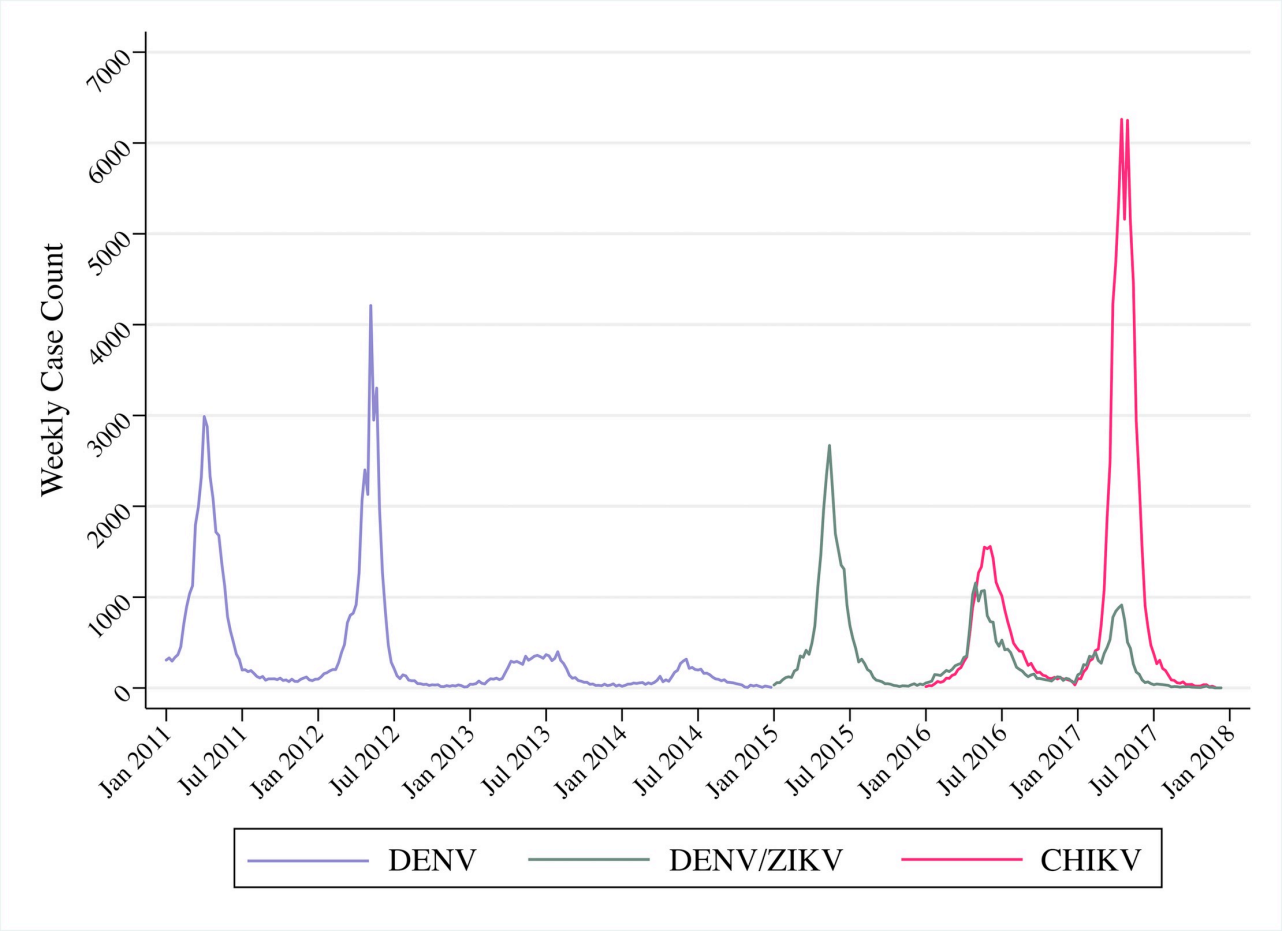
Weekly dengue, dengue/Zika, and chikungunya cases in Fortaleza, 2011–2017. Dengue and chikungunya cases were reclassified in 2016 and 2017, and dengue cases were considered indistinguishable from Zika cases from 2015–2017.

A total of 213,573 arbovirus cases were confirmed in Fortaleza during the study period. Table 1 presents total yearly case counts of each virus after confirmed cases were reclassified to account for misdiagnosis during 2016–2017, and dengue cases were merged with Zika cases during 2015–2017. The epidemic years 2011 and 2012 were marked by the introduction or reintroduction of DENV serotypes (DENV-1 in 2011 and DENV-4 in 2012). These two serotypes predominated during the interepidemic years 2013 and 2014. A DENV/ZIKV epidemic occurred in 2015, followed by two epidemic waves of CHIKV in 2016 and 2017. In 2017 alone, the CHIKV epidemic had nearly double the cases of the previous largest epidemic of DENV in 2011. The epidemiological pattern of CHIKV in 2016 and 2017 was opposite to that of the novel introduction of DENV-4 in 2012 and 2013, with a stronger second epidemic wave. In addition, the epidemiological curve for DENV/ZIKV appeared to peak earlier than that of CHIKV in 2016 (Fig 3).

**Table 1:** Confirmed dengue, dengue/Zika, and chikungunya cases in Fortaleza, 2011–2017. Dengue and chikungunya cases were reclassified in 2016 and 2017, and dengue cases were considered indistinguishable from Zika cases from 2015–2017.

Arbovirus Type	2011	2012	2013	2014	2015	2016	2017	Total
DENV	33,695	30,194	8,583	4,847				77,319
DENV/ZIKV					25,925	16,585	10,180	52,690
CHIKV						22,909	60,655	83,564
**Total**	33,695	30,194	8,583	4,847	25,925	39,494	70,835	213,573

**Fig 4:**
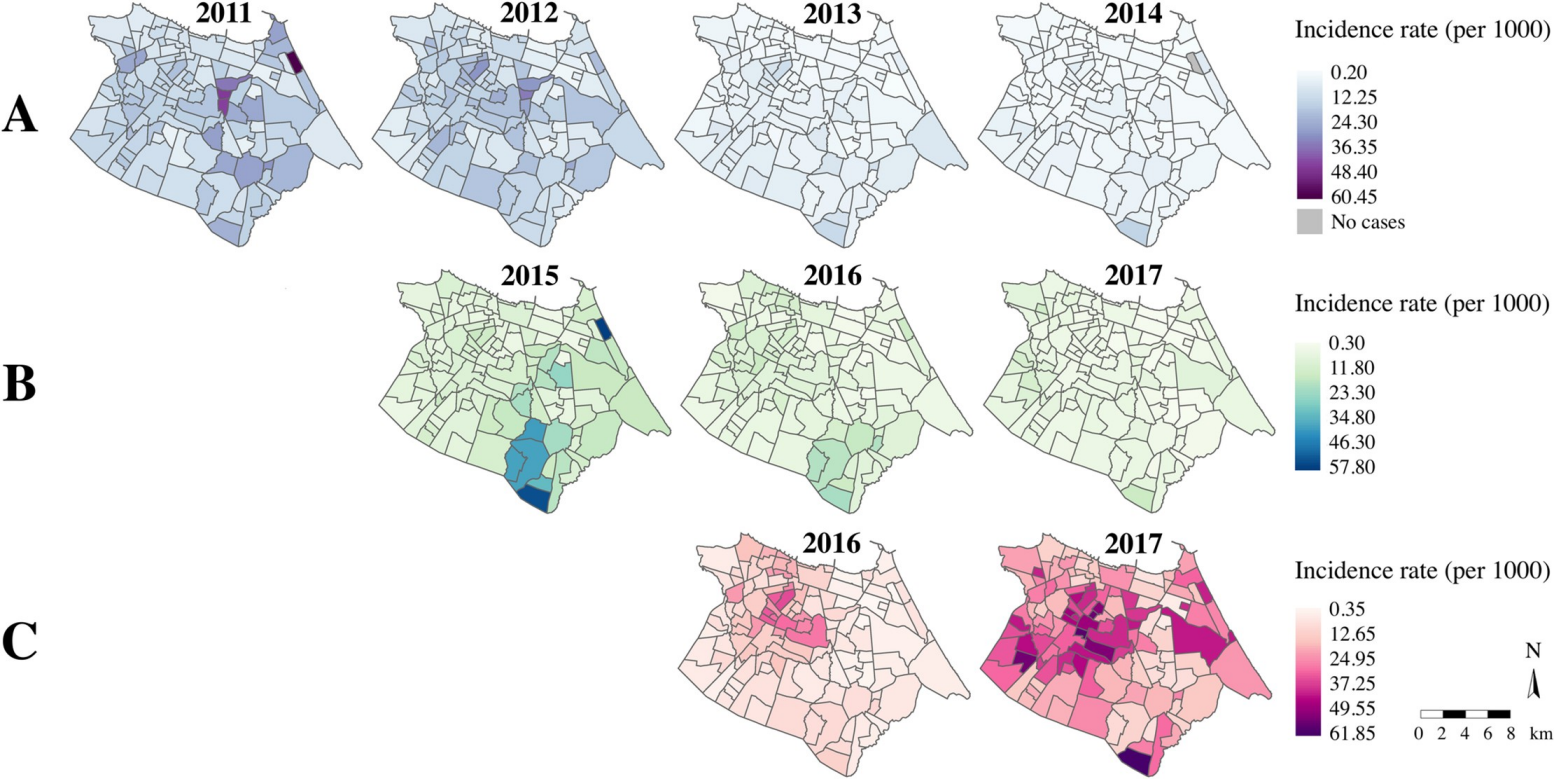
DENV, DENV/ZIKV, and CHIKV incidence rates (per 1,000 people) by *bairro*, Fortaleza, 2011–2017. *A*: DENV incidence rates; *B*: DENV/ZIKV incidence rates; *C*: CHIKV incidence rates. Dengue and chikungunya cases were reclassified in 2016 and 2017, and dengue cases were considered indistinguishable from Zika cases from 2015–2017. Maps were created in R v.3.5.1 using georeferenced cases from SIMDA.

The spatial spread of DENV, DENV/ZIKV, and CHIKV during each year of the study period is presented in Fig 4. While the DENV epidemics in 2011 and 2012 appeared distributed relatively evenly across the city, the DENV/ZIKV epidemics in 2015 and 2016 demonstrated substantial spatial heterogeneity, with a particularly high proportion of cases in the southern *bairros* of the city. As described previously, DENV was concentrated around the periphery of the city during the interepidemic years of 2013 and 2014 [13]. Unlike the large DENV epidemics in 2011 and 2012, the first epidemic wave of CHIKV was concentrated in the center-west *bairros* of the city.

**Fig 5:**
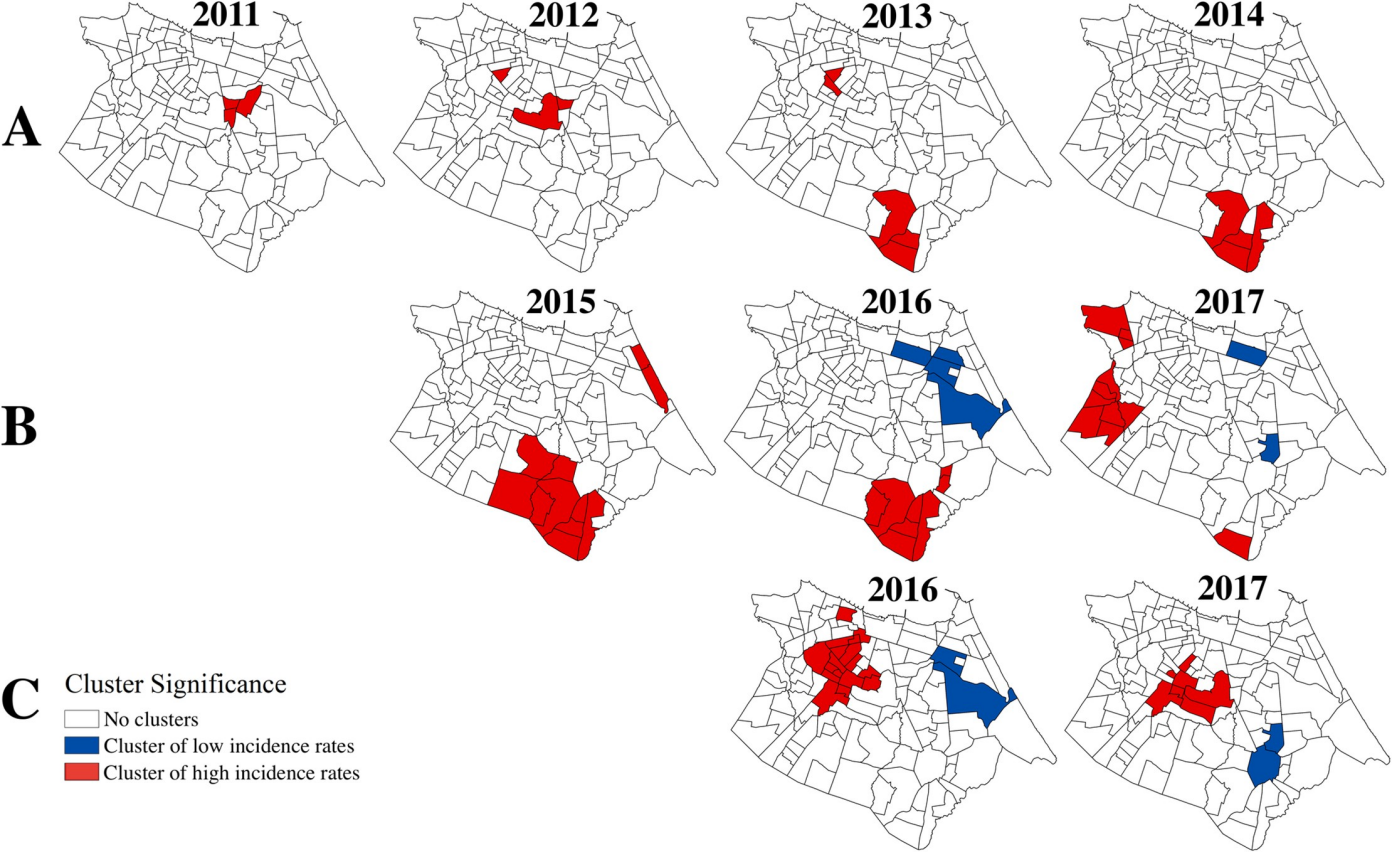
DENV, DENV/ZIKV, and CHIKV clusters by *bairro*, Fortaleza, 2011–2017. *A*: DENV clusters; *B*: DENV/ZIKV clusters; *C*: CHIKV clusters. Dengue and chikungunya cases were reclassified in 2016 and 2017, and dengue cases were considered indistinguishable from Zika cases from 2015–2017. Clusters of georeferenced cases from SIMDA were detected and visualized in ArcMap v.10.3 (ESRI, Redlands, CA, USA).

The local G* statistic revealed significant high clusters of incidence rates of each of the viruses during each year of the study period (Fig 5). Some areas exhibited persistent clustering for the same virus(es) over several years, such as the southern *bairros* for DENV and DENV/ZIKV from 2013 to 2016, and the center-west *bairros* for CHIKV from 2016 to 2017. DENV incidence rates showed minimal clustering in a few central *bairros* from 2011 and 2012, in agreement with the diffuse transmission pattern of DENV during these epidemic years (Fig 4). The center-west *bairros* that comprised the CHIKV clusters in 2016 and 2017 were almost entirely distinct from the *bairros* that comprised DENV or DENV/ZIKV clusters in previous years. Notably, there was no overlap of significant clusters of high incidence rates during the period when DENV/ZIKV and CHIKV co-circulated.

**Fig 6:**
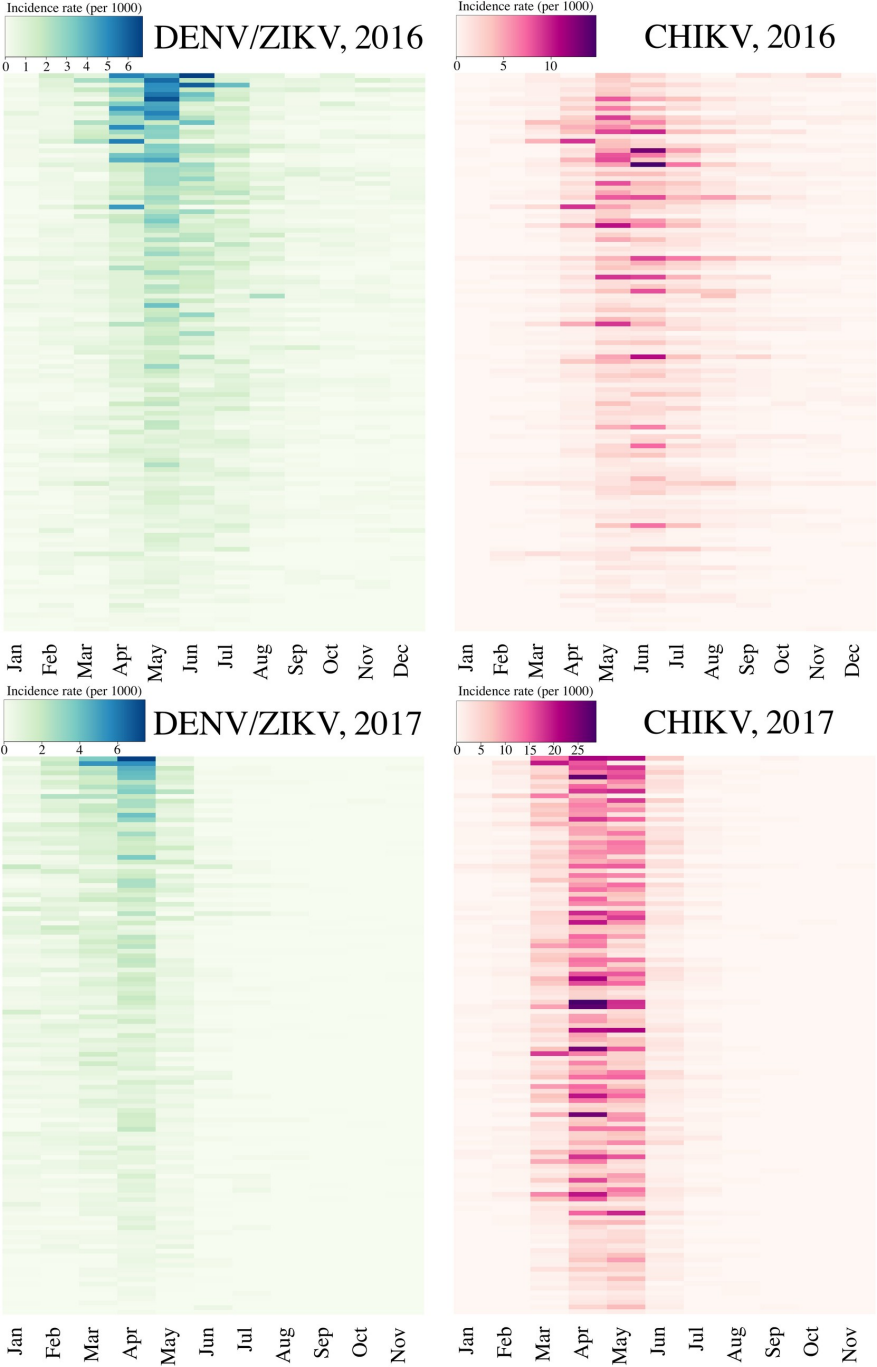
Heat maps showing incidence rates by month and *bairro* for DENV/ZIKV and CHIKV in 2016 and 2017. *Bairros* were sorted in decreasing order of each year’s mean incidence rate of DENV/ZIKV. Dengue and chikungunya cases were reclassified, and dengue cases were considered indistinguishable from Zika cases.

Indeed, heat maps of the monthly incidence rates of each of the viruses by *bairros* (Fig 6) showed that *bairros* with higher monthly DENV/ZIKV incidence rates did not align to those with higher monthly CHIKV incidence rates in 2016. This pattern was not observed in 2017 when high CHIKV incidence rates were more uniform across *bairros*. Conversely, many *bairros* with low DENV/ZIKV incidence rates experienced high CHIKV incidence rates during peak transmission months in both years. However, even in *bairros* with high incidence rates for both DENV/ZIKV and CHIKV, high CHIKV incidence rates persisted later into the year than high DENV/ZIKV incidence rates.

**Fig 7:**
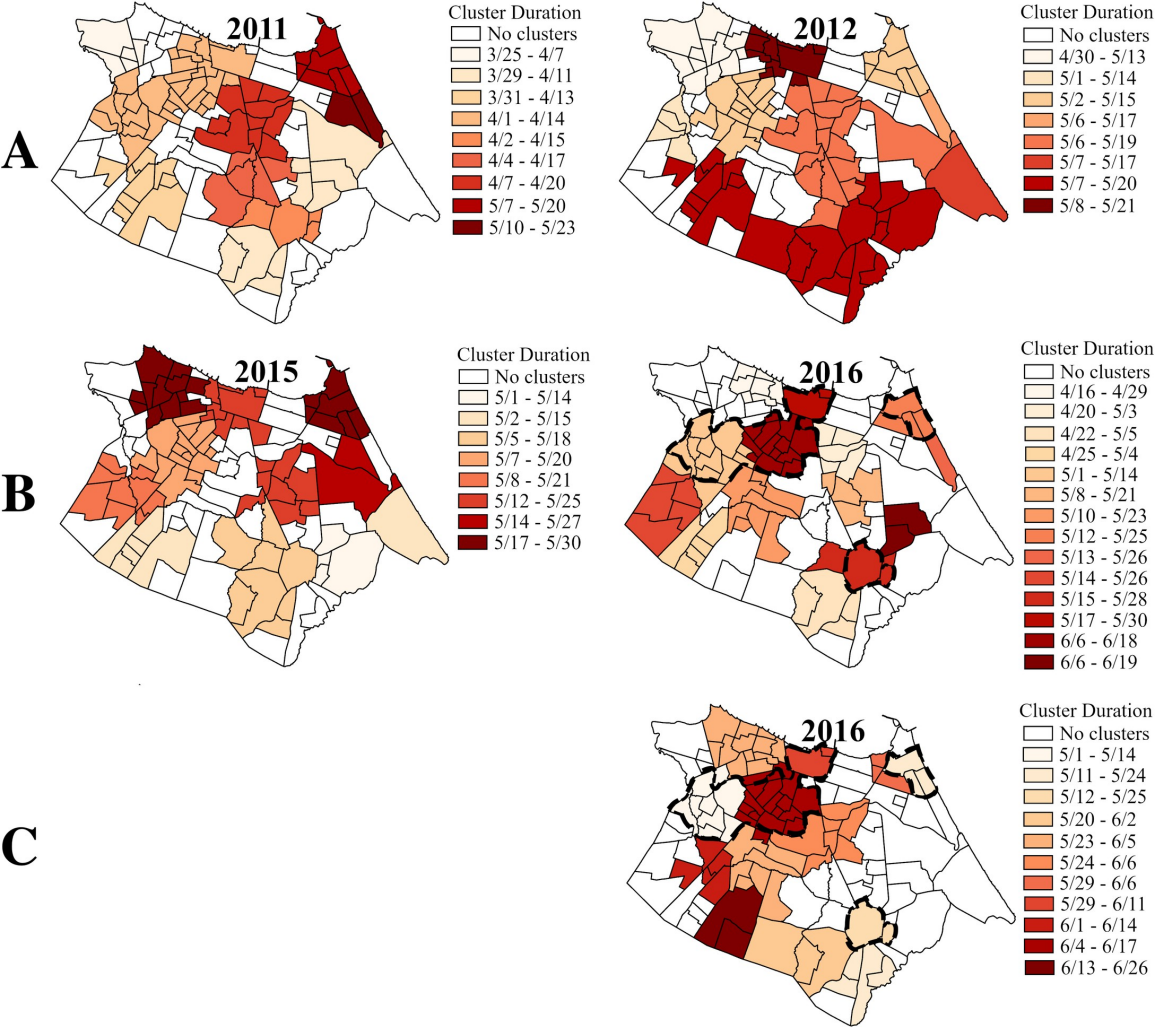
Space-time clusters of DENV, DENV/ZIKV, and CHIKV during years of introduction or reintroduction of new serotypes or viruses. *A*: DENV; *B*: DENV/ZIKV; *C*: CHIKV. Clusters are colored from light to dark according to when each cluster was observed (the earliest clusters are the lightest). The legend indicates the month/day of the start and end of the cluster. Dotted lines indicate *bairros* with any spatiotemporally overlapping DENV/ZIKV and CHIKV clusters with more than one day overlap. Dengue and chikungunya cases were reclassified in 2016 and 2017, and dengue cases were considered indistinguishable from Zika cases from 2015–2017. Space-time clusters of georeferenced cases from SIMDA were detected in SaTScan v.9.6 and visualized in ArcMap v.10.3 (ESRI, Redlands, CA, USA).

The space-time scan statistic was applied to each year to compare the clustering pattern of each of the viruses across epidemic years of introduction or reintroduction of new serotypes or viruses (Fig 7) and years of interepidemic or continued transmission (S2 Fig). Nearly all observed clusters occurred within the high-transmission season of the respective year. In the large epidemic years of 2012 and 2015, all DENV or DENV/ZIKV clusters occurred within a one-month period. In contrast, clusters stretched over almost two months during the CHIKV epidemics in 2016 and 2017. In the interepidemic years 2013, 2014, and 2016, DENV or DENV/ZIKV clusters were distributed over two to four months. Across all years, no singular spatial diffusion pattern was observed; the location of the earliest and latest clusters observed varied year to year.

Importantly, there was minimal spatiotemporal overlap between DENV/ZIKV and CHIKV clusters in 2016 and 2017. In the first two weeks of May 2016, clusters of both DENV/ZIKV and CHIKV were observed in some northwestern *bairros* of Fortaleza (Fig 7). Additional overlap in the second half of May 2016 was present in three center-south *bairros*, and in three northeastern *bairros*. There was no clustering of CHIKV in the southern tip of the city, where the highest incidence rates of DENV occurred, until the second half of May 2016, when all DENV/ZIKV clusters had subsided. In April 2017, there was again overlap between DENV/ZIKV and CHIKV clusters in some northern and southeastern *bairros* (S2 Fig). However, the CHIKV clusters covering most of the area of the city occurred in May 2017, after the last DENV/ZIKV clusters were observed.

**Fig 8:**
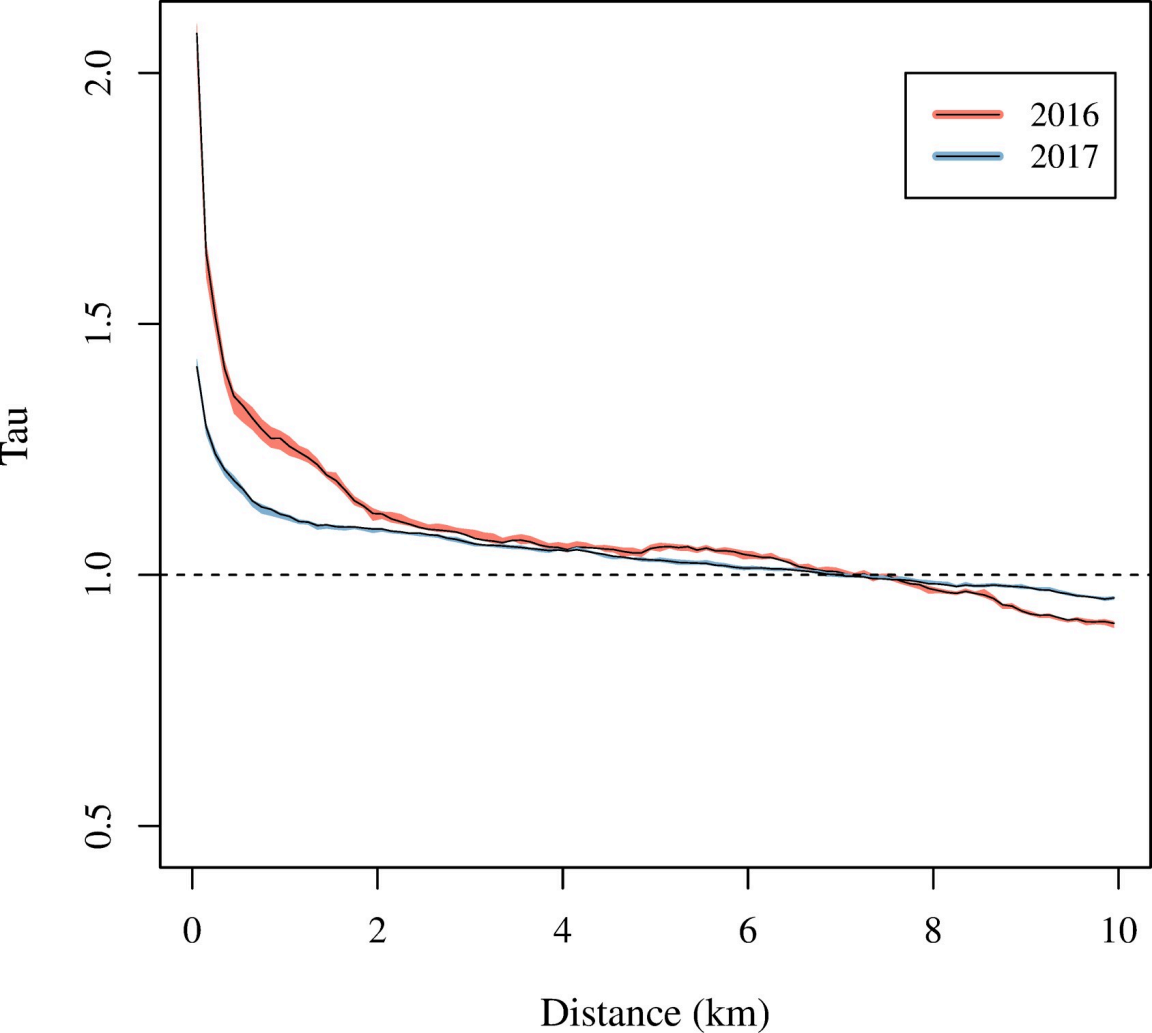
Spatiotemporal dependence analysis of dengue/Zika and chikungunya cases in 2016 and 2017. Tau (τ) represents the increase in the probability that a case is the same virus type as another case occurring within two weeks and at a certain distance range from it, relative to what would be expected had there been no spatiotemporal dependence.

**Fig 9:**
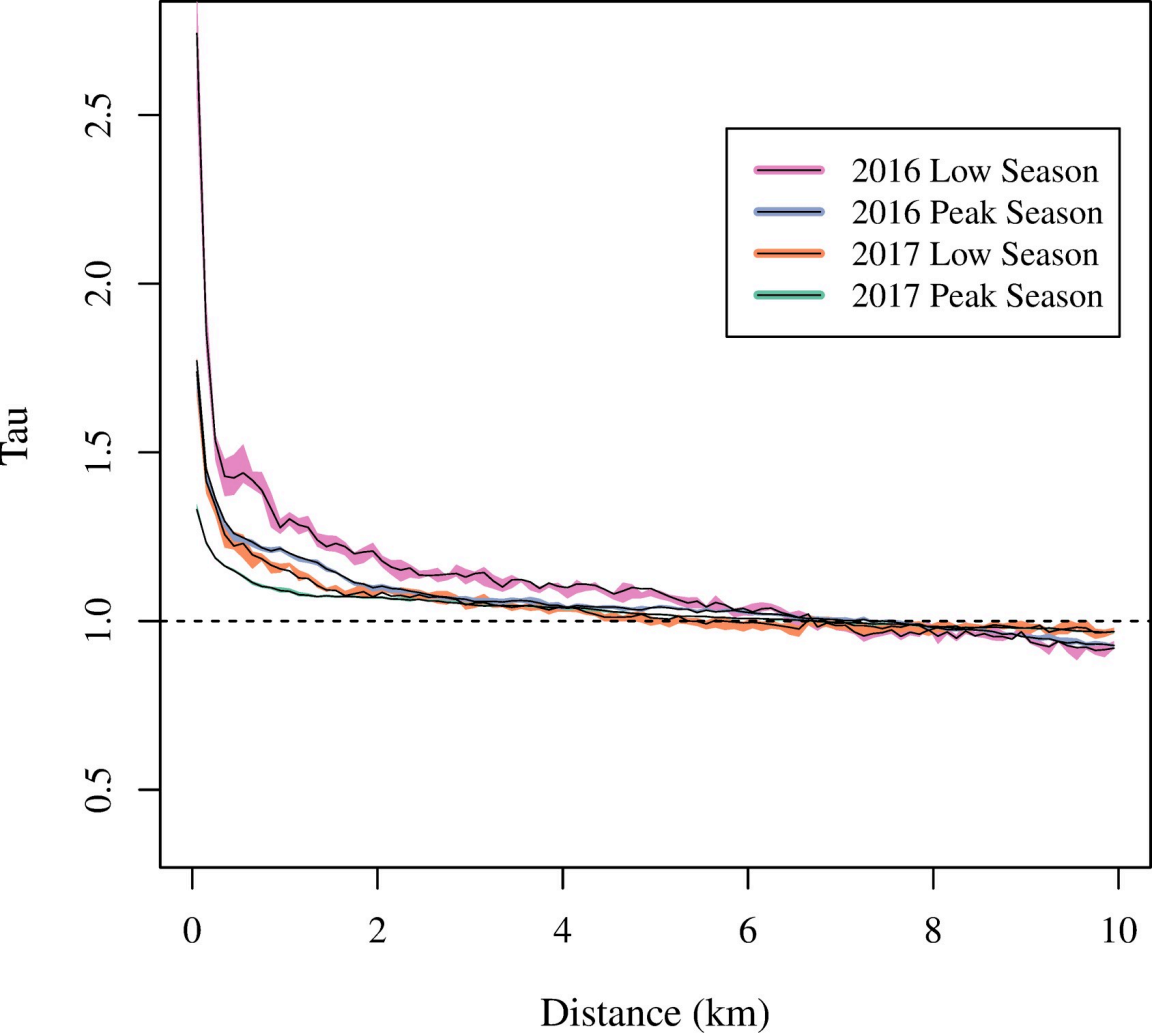
Spatiotemporal dependence analysis of dengue/Zika and chikungunya cases during low and peak seasons in 2016 and 2017. Tau (τ) represents the increase in probability that a case is the same virus type as another case occurring within two weeks and at a certain distance range from it, relative to what would be expected had there been no spatiotemporal dependence. Peak seasons: April-July in 2016 and March-May in 2017. Remaining months were low seasons.

A spatiotemporal dependence analysis was conducted to assess whether DENV/ZIKV and CHIKV transmitted independently of one another in 2016 and 2017 (Figs 8 and 9). In yearly and seasonal analyses, the τ-statistic was significantly greater than one at distances of up to 6.8 km (*p*<0.05), indicating more clustering of cases of the same virus than would be expected had the cases been randomly distributed over space within two-week intervals of time. In sensitivity analyses using original, non-reclassified data, the τ-statistic remained significantly greater one at distances of up to 6.6 km (S3 and S4 Figs).

Although significant spatiotemporal dependence was observed in both years, the τ-statistic was larger in 2016 compared to 2017 (Fig 8). In 2016, there was a 1.36-fold (95% CI: 1.32, 1.37) increase in the probability that a case occurring 400–500 m away from and within two weeks of another case was the same virus. In 2017, there was a smaller but still significant 1.19-fold (95% CI: 1.18, 1.20) increase in the probability of two cases being the same virus type within the same time and distance interval. The increase in probability fell to 1.08 (95% CI: 1.08, 1.09) for cases occurring 2.9–3 km from one another in 2016 and to 1.07 (95% CI: 1.06, 1.07) in 2017 for the same time and distance interval. However, both yearly estimates remained significantly greater than one at distances of up to 6.8 km.

Spatiotemporal dependence analysis was also conducted separately for the low and peak transmission seasons of each year (Fig 9). The τ-statistic was significantly greater than one for both seasons in both years at distances of up to 4.4 km (*p*<0.05). Interestingly, the low seasons of each year exhibited higher spatiotemporal clustering than the peak seasons. For example, at distances of 400-500m, the relative probability of a pair of cases being the same type was 1.42 (95% CI: 1.37, 1.49) during the low season in 2016 and 1.26 (95% CI: 1.24, 1.26) during the peak season in 2016.

## Discussion

This paper assessed the transmission dynamics of three co-circulating arboviruses in Fortaleza, Brazil. We used retrospective surveillance data from 2011 to 2017 that allowed comparison of transmission patterns before and after multiple viruses began co-circulating in the city. Our results show that the relative risk for each arbovirus was not evenly distributed across the city when multiple viruses co-circulated. Although we were not able to distinguish between DENV and ZIKV, the two viruses together showed space-time clustering patterns distinct from CHIKV when all three viruses were transmitting in Fortaleza. We also illustrated important differences between the pattern of spread of CHIKV and that of the previous introduction of new DENV serotypes.

Although there is no definitive explanation for the clustered transmission dynamics we observed, we evaluate a few potential hypotheses. Contextual factors, such as environmental conditions and population density, are important determinants of transmission, but they are likely to affect all three viruses in similar ways, and so are unlikely to explain distinct viral clustering patterns. Thus, the simplest explanation for our findings is that focal transmission patterns are inherent to arboviruses. Clustering at distances below 100 m may reflect direct transmission, considering the restricted flight range of the *Ae*. *aegypti* mosquito [58]. Clustering at greater distances might arise if viruses happen to begin circulating in isolated spatial areas and do not spread far beyond them over time. We provide several reasons to call this explanation into doubt. First, we observed significant spatiotemporal clustering at unusually large distances (up to 6.8 km) in addition to distinct spatial clusters of yearly incidence rates. A previous study in Thailand showed no spatiotemporal dependence between different serotypes of DENV at distances greater than 1.8 km and insignificant dependence at distances between 0.7 km and 1.8 km [17]. Second, considering human mobility in an urban setting, we would expect infected individuals to regularly introduce viruses to even distant areas within the same city. Third, prior introductions or reintroductions of arboviruses (DENV-1 in 2011 and DENV-4 in 2012) did not show substantial yearly spatial clustering patterns, even though space-time clusters occurred within a narrower timeframe than CHIKV in 2016.

These results open the possibility for processes complementary to direct transmission that may account for the observed spatiotemporal clustering patterns. For example, a competitive advantage for DENV or ZIKV in dually infected mosquitoes could have prevented CHIKV from spreading to the southern *bairros* where DENV/ZIKV dominated in 2016. However, the contradictory existing literature on the topic of competitive suppression in *Aedes* mosquitoes leaves this question open [18–21]. A recent review underscored the dearth of studies evaluating the effect of sequential infection on virus transmission potential; most studies of competitive suppression have been performed on simultaneously infected mosquitoes [59]. Even fewer studies have assessed the potential for competitive suppression in co-infected humans [60,61]. The immunity profiles of the population might also affect where arboviruses transmit if protective or enhancing immunological cross-reactions exist. If immunity profiles are spatially clustered, then the transmission of viruses in subsequent years could also be spatially clustered due to immunological interactions. In our case, such interactions are unlikely to explain the transmission dynamics of CHIKV, which as an alphavirus is antigenically distinct from and unlikely to react with flavivirus antibodies.

Besides potential interactions between viruses, intrinsic differences between them or seasonal variation across years may contribute to the observed transmission dynamics. For instance, the introduction of CHIKV in 2016 may not be comparable to the introduction or reintroduction of DENV serotypes in 2011 and 2012 if the viruses differ in their capacity to infect or be transmitted by mosquitoes. Differences in clustering patterns during these years might be attributable to viral differences, rather than viral interactions. Unfortunately, conflicting vector competence studies do not provide a resolution to this question [23–25]. Climatological factors may also render different years incomparable. A previous study on DENV transmission in Fortaleza found that transmission persists longer into the year during interepidemic years [13]. In agreement with this finding, we found that space-time clusters extended over longer periods during DENV interepidemic years compared to DENV and DENV/ZIKV epidemic years from 2011–2015. However, the temporal distribution of space-time clusters during CHIKV epidemic years more closely resembled the distribution during DENV interepidemic years. Although co-circulation may have extended the occurrence of CHIKV epidemic clusters compared to DENV epidemic clusters, we cannot rule out the impact of seasonal variation across years.

Our finding that arboviruses display distinct space-time clustering patterns adds to the mixed and sparse existing literature on this topic. One recent study used space-time scan statistics to investigate clustering patterns of simultaneous dengue, Zika, and chikungunya epidemics in Rio de Janeiro [62]. With some exceptions, the authors of this study did not observe clusters of the same diseases in the same place and time, similar to our findings [62]. Two previous studies, which used global statistics or clustering tests to assess overlap between co-circulating viruses, found substantial co-occurrence of multiple viruses in the same neighborhoods [63,64]. One study in Merida, Mexico, found high agreement between incidence rates of co-circulating CHIKV and ZIKV by census tract using a measure of spatiotemporal coherence; however, local spatial clusters of each virus did not overlap [63]. Another study in Colombia reported spatiotemporal overlap between CHIKV and DENV using univariate and multivariate space-time clusters [64]. Differences between studies, while potentially attributable to actual differences in transmission dynamics, might instead arise from differences in analytical techniques. Measures of spatiotemporal coherence or space-time clustering tests may be ill-equipped to capture clustering patterns that occur at specific spatial and temporal scales. In addition, since these studies also relied on surveillance data without laboratory confirmation of clinical diagnoses, misdiagnosis may be a common challenge.

To our knowledge, only the Merida study also compared the spatiotemporal dynamics of multiple arboviruses longitudinally [63], and observed high spatiotemporal coherence of all viruses across the years. While CHIKV clusters occurred in an area of historical DENV clustering, ZIKV clusters did not coincide with areas where DENV circulated in previous years. In our study, the lack of a specific serological test for DENV and ZIKV in the context of their co-circulation prevented us from distinguishing between the two flaviviruses [47]. The observed DENV/ZIKV clustering in the south of Fortaleza in 2015 and 2016 may be explained by previous DENV transmission in those areas in 2013 and 2014, but we were unable to discern the direction of this effect. For future studies that have the capacity to differentiate between the two viruses, it would be important to assess how immunity from previous DENV infections could affect future ZIKV infections at the neighborhood level. Two recent cohort studies have suggested that historical DENV infection could protect from symptomatic ZIKV infection, while recent DENV infection could increase the likelihood of ZIKV infection [29,31].

This study has some limitations. First, although we aimed to mitigate the low fidelity of confirmed dengue diagnoses in 2016 and 2017 by estimating and reclassifying the proportion of dengue cases misdiagnosed as chikungunya cases, we have no reliable way to test the validity of our assumptions. We applied the reclassification algorithm globally, assuming that misdiagnosis rates were equal across *bairros*. However, it is possible that *bairros* with more chikungunya cases had more misdiagnosis of dengue cases; alternatively, those *bairros* that reported more chikungunya cases may have had lower rates of dengue misdiagnosis, perhaps due to heightened vigilance and proper identification of chikungunya cases. We lacked data that would allow us to make any substantiated assumptions about neighborhood-level classification issues. Nevertheless, we showed that clustering patterns of dengue/Zika and chikungunya cases remained distinct even when the spatial range of chikungunya was assumed to extend beyond the original cases. Reclassifying dengue cases based on neighborhood-level chikungunya incidence would only heighten existing clustering patterns and impose a spatial pattern to the analysis. Thus, we consider our reclassification algorithm to be the most conservative and appropriate approach to evaluate spatiotemporal overlap between dengue/Zika and chikungunya cases.

A second limitation is that we were unable to assess the incidence of co-infected cases, who may present with non-specific symptoms that increase the likelihood of misdiagnosis [4]. In fact, no case of co-infection was reported in any year, reflecting a limitation of the surveillance effort. Third, and common to all surveillance studies, data are only available for cases that present symptoms. Fourth, there may be bias in our spatial clustering analyses because of underreporting among higher-income *bairros*. In those areas, people may use private health centers (instead of the public health system), and those do not report the totality of cases to SINAN [65]. However, our comparisons of clustering patterns over time would not be biased unless there were changes in case reporting rates across *bairros* over time. Importantly, underlying spatial heterogeneity in measurement or changes in measurement over time would not affect the validity of our spatiotemporal dependence analysis, since all arboviruses would be equally affected by such confounding factors in theory.

Our results suggest that the risk of arboviruses is not uniform across small spatial and temporal scales within a city, even if multiple arboviruses circulate at the same time within that city. Surveillance and vector control efforts could be better targeted if spatiotemporal clusters of individual arboviruses were identified and addressed directly in real-time. Enhanced physician awareness of arbovirus risk across specific *bairros* over time might also improve diagnosis and care. Guided by refined epidemiological criteria from surveillance officers, physicians may improve their diagnostic accuracy in the absence of expensive serological tests. Of course, the epidemiological context does not replace the need for specific serological tests to provide reliable measures of infection. Additional research using laboratory-confirmed case data has the potential to confirm the impact of co-circulation on the transmission dynamics of DENV, ZIKV, and CHIKV and to investigate potential long-term interactions between the viruses.

## Supporting information

S1 Fig *Bairros* of Fortaleza. A *bairro* name key is provided in S1 Table.
(TIF)

S2 Fig Space-time clusters of DENV, DENV/ZIKV, and CHIKV during non-introduction years. *A*: DENV; *B*: DENV/ZIKV; *C*: CHIKV. Clusters are colored from light to dark according to when each cluster was observed (the earliest clusters are the lightest). The legend indicates the month/day of the start and end of the cluster. Dotted lines indicate *bairros* with any spatiotemporally overlapping DENV/ZIKV and CHIKV clusters. DENV and chikungunya cases were reclassified in 2016 and 2017, and DENV was considered indistinguishable from ZIKV from 2015–2017. Space-time clusters of georeferenced cases from SIMDA were detected in SaTScan v.9.6 and visualized in ArcMap v.10.3 (ESRI, Redlands, CA, USA).
(TIF)

S3 Fig Spatiotemporal dependence analysis of DENV/ZIKV and chikungunya cases in 2016 and 2017 using original cases. Tau (τ) represents the increase in the probability that a case is the same virus type as another case occurring within two weeks and at a certain distance range from it, relative to what would be expected had there been no spatiotemporal dependence.
(TIF)

S4 Fig Spatiotemporal dependence analysis of DENV/ZIKV and chikungunya cases during low and peak seasons in 2016 and 2017 using original cases. Tau (τ) represents the increase in probability that a case is the same virus type as another case occurring within two weeks and at a certain distance range from it, relative to what would be expected had there been no spatiotemporal dependence. Peak seasons: April-July in 2016 and March-May in 2017. Remaining months were low seasons.
(TIF)

S1 Table *Bairro* identification numbers in Fortaleza. (PDF)

S2 Table Number and proportion of dengue cases reclassified as CHIKV by *bairro* in 2016 and 2017. (PDF)
