# Supplementary figures and images for: Spatiotemporal transmission dynamics of co-circulating dengue, Zika, and chikungunya viruses in Fortaleza, Brazil: 2011–2017
Source: PLoS Negl Trop Dis. 2020 Oct 26;14(10):e0008760. doi: 10.1371/journal.pntd.0008760 (PMC7644107; doi:10.1371/journal.pntd.0008760)

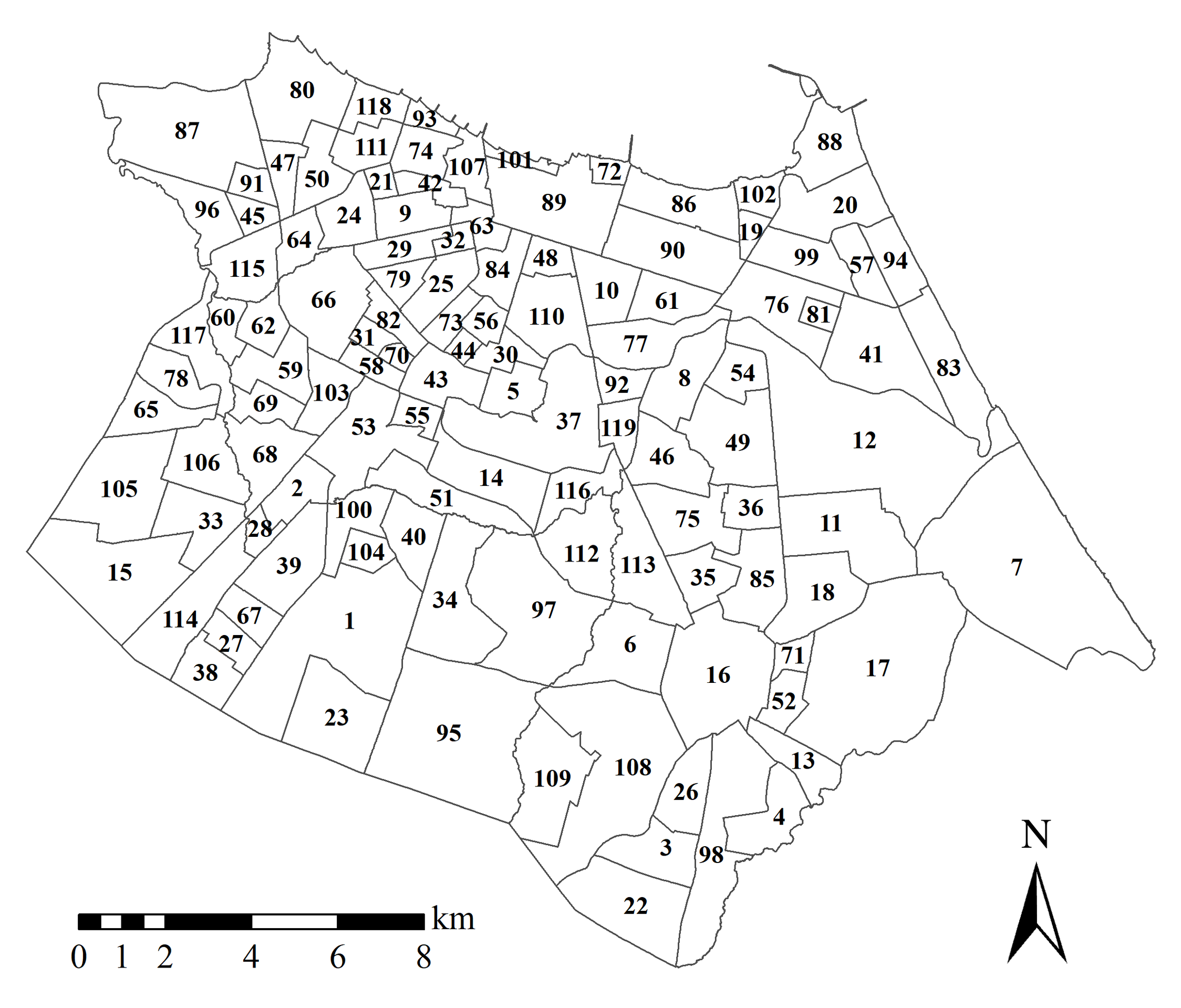

Supplement: S1 Fig — A bairro name key is provided in S1 Table. (TIF) [file pntd.0008760.s001.tif]

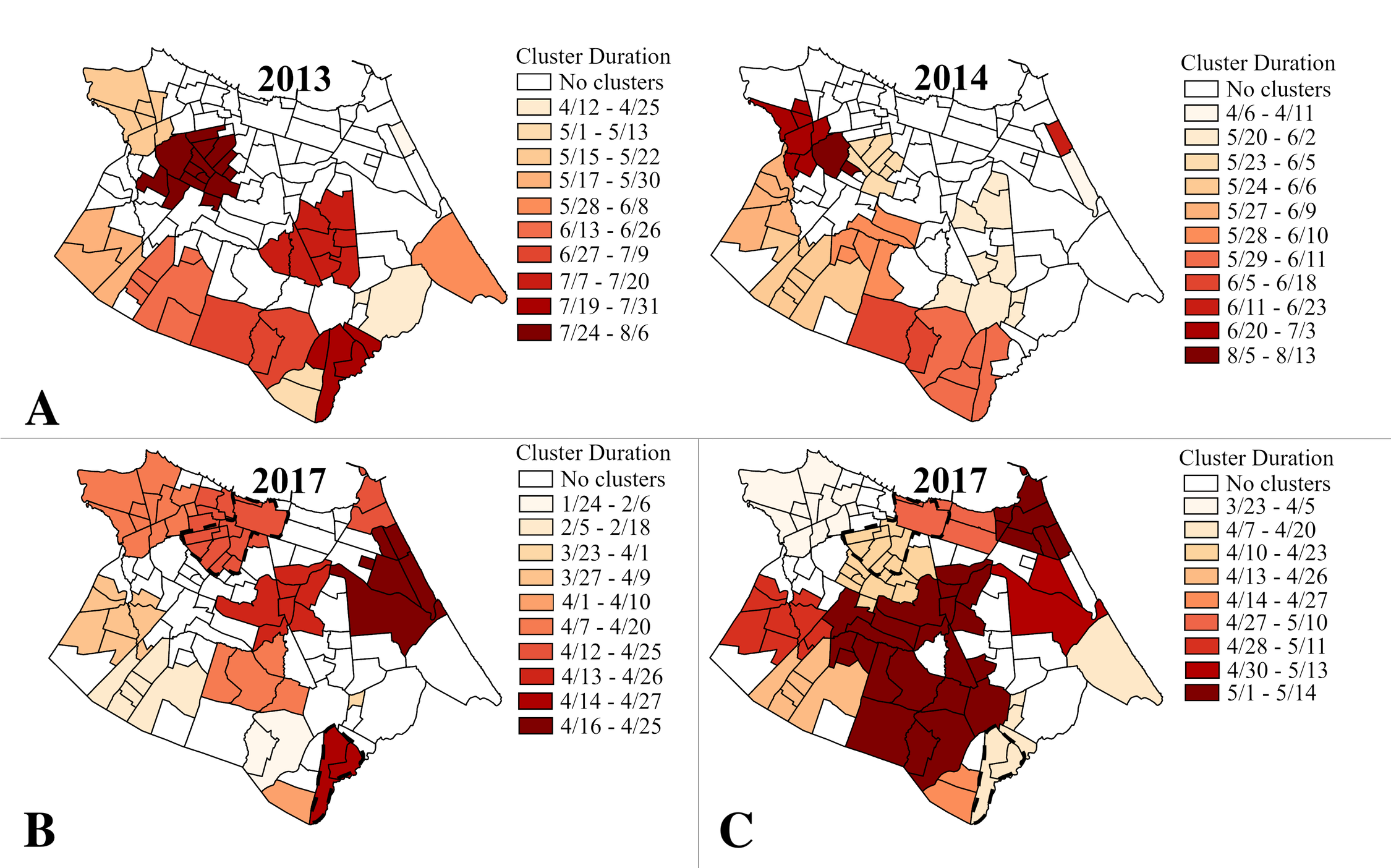

Supplement: S2 Fig — A: DENV; B: DENV/ZIKV; C: CHIKV. Clusters are colored from light to dark according to when each cluster was observed (the earliest clusters are the lightest). The legend indicates the month/day of the start and end of the cluster. Dotted lines indicate bairros with any spatiotemporally overlapping DENV/ZIKV and CHIKV clusters. DENV and chikungunya cases were reclassified in 2016 and 2017, and DENV was considered indistinguishable from ZIKV from 2015–2017. Space-time clusters of georeferenced cases from SIMDA were detected in SaTScan v.9.6 and visualized in ArcMap v.10.3 (ESRI, Redlands, CA, USA). (TIF) [file pntd.0008760.s002.tif]

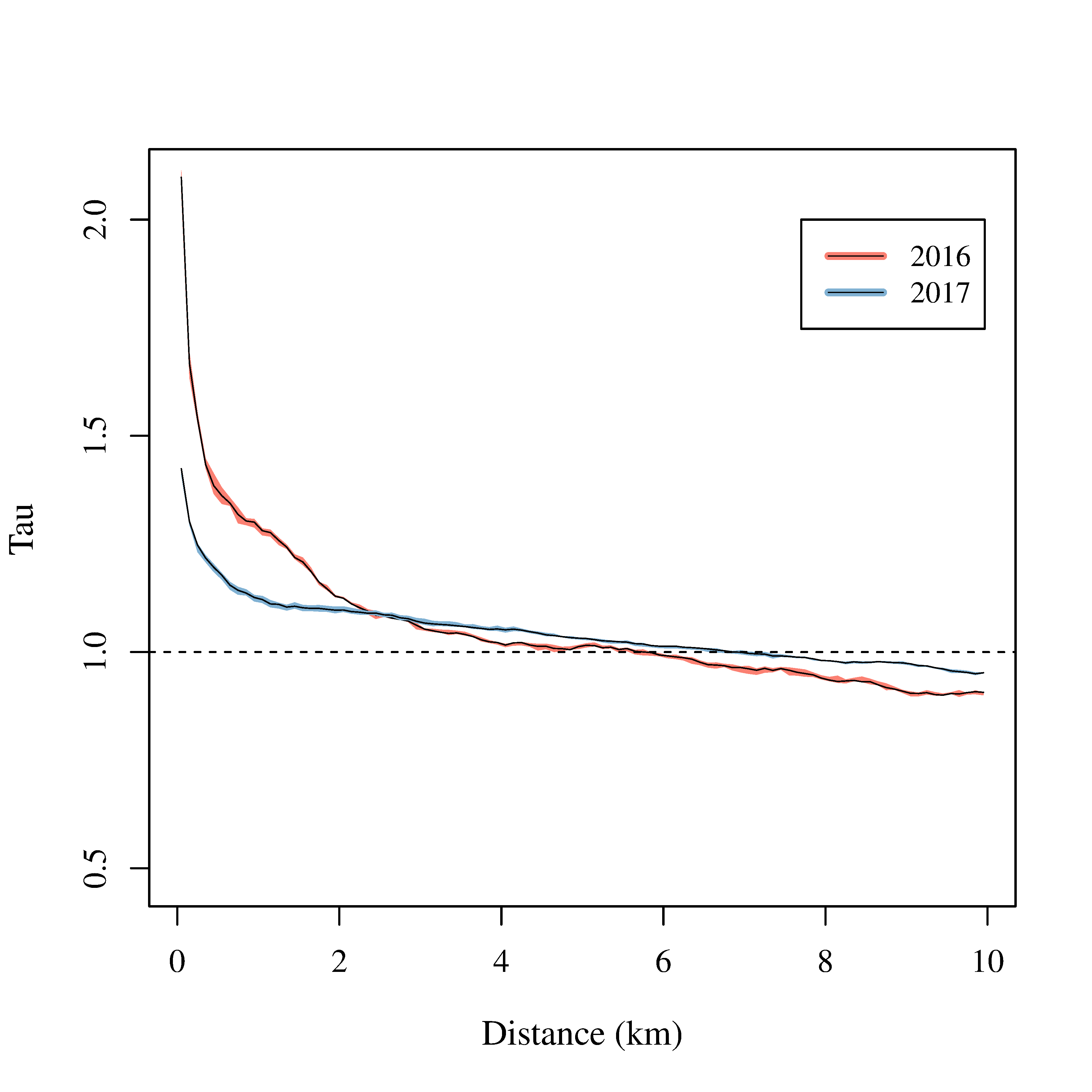

Supplement: S3 Fig — Tau (τ) represents the increase in the probability that a case is the same virus type as another case occurring within two weeks and at a certain distance range from it, relative to what would be expected had there been no spatiotemporal dependence. (TIF) [file pntd.0008760.s003.tif]

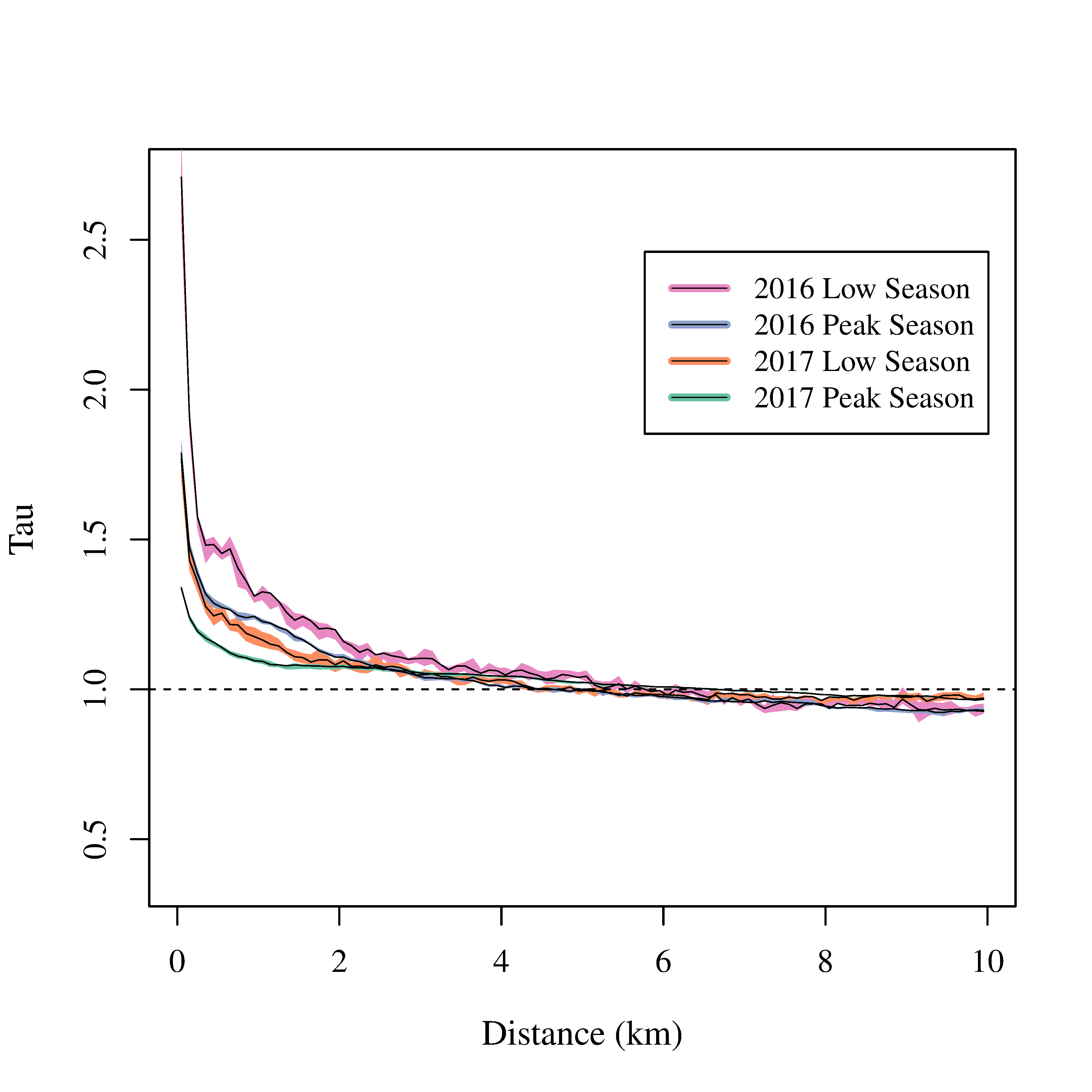

Supplement: S4 Fig — Tau (τ) represents the increase in probability that a case is the same virus type as another case occurring within two weeks and at a certain distance range from it, relative to what would be expected had there been no spatiotemporal dependence. Peak seasons: April-July in 2016 and March-May in 2017. Remaining months were low seasons. (TIF) [file pntd.0008760.s004.tif]
