# Supplementary material for: Spatiotemporal transmission dynamics of co-circulating dengue, Zika, and chikungunya viruses in Fortaleza, Brazil: 2011–2017
Source: PLoS Negl Trop Dis. 2020 Oct 26;14(10):e0008760. doi: 10.1371/journal.pntd.0008760 (PMC7644107; doi:10.1371/journal.pntd.0008760)
Supplement: S1 Table — (PDF) [file pntd.0008760.s005.pdf]

S1 Table. ***Bairro*** **identification numbers in Fortaleza.**

ID	*Bairro*
1	Mondubim
2	Vila Pery
3	Ancuri
4	São Bento
5	Vila União
6	Barroso
7	Sabiaguaba
8	Salinas
9	São Gerardo
10	Joaquim Távora
11	Sapiranga Coité
12	Edson Queiroz
13	Coaçu
14	Serrinha
15	Siqueira
16	Messejana
17	Lagoa Redonda
18	José de Alencar
19	Varjota
20	Vicente Pinzon
21	Vila Ellery
22	Pedras
23	Planalto Ayrton Senna
24	Presidente Kennedy
25	Rodolfo Teófilo
26	Parque Santa Maria
27	Parque Santa Rosa
28	Parque São José
29	Parquelândia
30	Parreão
31	Pan Americano
32	Parque Araxá
33	Bom Jardim
34	Parque dois Irmãos
35	Parque Iracema
36	Parque Manibura
37	Aeroporto
38	Parque Presidente Vargas
39	Manoel Sátiro
40	Dendê
41	Manuel Dias Branco
42	Monte Castelo
43	Montese
44	Bom Futuro
45	Olavo Oliveira
46	Jardim das Oliveiras
47	Jardim Iracema
48	José Bonifácio
49	Luciano Cavalcante
50	Floresta
51	Itaperi
52	Guajeru
53	Parangaba
54	Guararapes
55	Itaoca
56	Jardim América
57	De Lourdes
58	Demócrito Rocha
59	Henrique Jorge
60	Autran Nunes

ID	*Bairro*
61	Dionísio Torres
62	Dom Lustosa
63	Farias Brito
64	Padre Andrade
65	Conjunto Ceará II
66	Pici
67	Conjunto Esperança
68	Bonsucesso
69	João XXIII
70	Couto Fernandes
71	Curió
72	Praia de Iracema
73	Damas
74	Carlito Pamplona
75	Cidade dos Funcionarios
76	Cocó
77	São João do Tauape
78	Conjunto Ceará I
79	Amadeu Furtado
80	Barra do Ceará
81	Cidade 2000
82	Bela Vista
83	Praia do Futuro II
84	Benfica
85	Cambeba
86	Meireles
87	Vila Velha
88	Cais do Porto
89	Centro
90	Aldeota
91	Jardim Guanabara
92	Alto da Balança
93	Pirambú
94	Praia do Futuro I
95	Prefeito José Walter
96	Quintino Cunha
97	Passaré
98	Paupina
99	Papicu
100	Maraponga
101	Moura Brasil
102	Mucuripe
103	Jóquei Clube
104	Jardim Cearense
105	Granja Lisboa
106	Granja Portugal
107	Jacarecanga
108	Jangurussu
109	Palmeiras
110	Fátima
111	Álvaro Weyne
112	Boa Vista
113	Cajazeiras
114	Canindezinho
115	Antônio Bezerra
116	Dias Macedo
117	Genibaú
118	Cristo Redentor
119	Aerolândia
