# Supplementary material for: Spatiotemporal transmission dynamics of co-circulating dengue, Zika, and chikungunya viruses in Fortaleza, Brazil: 2011–2017
Source: PLoS Negl Trop Dis. 2020 Oct 26;14(10):e0008760. doi: 10.1371/journal.pntd.0008760 (PMC7644107; doi:10.1371/journal.pntd.0008760)
Supplement: S2 Table — (PDF) [file pntd.0008760.s006.pdf]

S2 Table. Number and proportion of DENV cases reclassified as CHIKV by *bairro* in 2016 and 2017.

Bairro	Confirmed DENV cases in 2016	DENV cases reclassified as CHIKV in 2016	Percentage reclassified in 2016	Confirmed DENV cases in 2017	DENV cases reclassified as CHIKV in 2017	Percentage reclassified in 2017
Aerolândia	99	30	30.3	88	21	23.86
Aeroporto	84	29	34.52	45	11	24.44
Aldeota	99	24	24.24	66	18	27.27
Alto da Balança	89	17	19.1	115	33	28.7
Álvaro Weyne	223	56	25.11	177	33	18.64
Amadeu Furtado	115	25	21.74	62	18	29.03
Ancuri	131	38	29.01	55	14	25.45
Antônio Bezerra	413	103	24.94	91	28	30.77
Autran Nunes	271	87	32.1	106	24	22.64
Barra do Ceará	803	246	30.64	351	73	20.8
Barroso	454	127	27.97	248	70	28.23
Bela Vista	159	44	27.67	59	10	16.95
Benfica	50	11	22	28	3	10.71
Boa Vista	81	26	32.1	83	19	22.89
Bom Futuro	50	14	28	32	8	25
Bom Jardim	334	103	30.84	415	100	24.1
Bonsucesso	475	133	28	357	91	25.49
Cais do Porto	89	27	30.34	81	17	20.99
Cajazeiras	76	23	30.26	52	11	21.15
Cambeba	41	6	14.63	6	0	0
Canindezinho	184	56	30.43	187	49	26.2
Carlito Pamplona	423	117	27.66	97	21	21.65
Centro	233	74	31.76	127	33	25.98
Cidade 2000	23	4	17.39	18	3	16.67
Cidade dos Funcionarios	74	22	29.73	39	10	25.64
Coaçu	70	14	20	11	1	9.09
Cocó	15	2	13.33	7	0	0
Conjunto Ceará I	264	78	29.55	286	76	26.57
Conjunto Ceará II	104	19	18.27	244	59	24.18
Conjunto Esperança	51	16	31.37	49	13	26.53
Couto Fernandes	70	16	22.86	13	2	15.38
Cristo Redentor	441	110	24.94	190	48	25.26
Curió	101	28	27.72	30	5	16.67
Damas	61	16	26.23	26	5	19.23
De Lourdes	4	1	25	2	1	50

Demócrito Rocha	131	35	26.72	36	6	16.67
Dendê	70	19	27.14	41	11	26.83
Dias Macedo	98	23	23.47	95	29	30.53
Dionísio Torres	20	5	25	12	2	16.67
Dom Lustosa	147	47	31.97	64	9	14.06
Edson Queiroz	80	20	25	214	45	21.03
Farias Brito	66	14	21.21	41	11	26.83
Fátima	57	18	31.58	29	7	24.14
Floresta	175	50	28.57	156	39	25
Genibaú	421	110	26.13	442	119	26.92
Granja Lisboa	268	60	22.39	353	85	24.08
Granja Portugal	341	87	25.51	560	128	22.86
Guajeru	169	47	27.81	18	3	16.67
Guararapes	3	1	33.33	3	0	0
Henrique Jorge	350	109	31.14	116	27	23.28
Itaoca	156	44	28.21	56	11	19.64
Itaperi	197	62	31.47	100	27	27
Jacarecanga	194	57	29.38	55	13	23.64
Jangurussu	1387	404	29.13	364	92	25.27
Jardim América	63	11	17.46	57	15	26.32
Jardim Cearense	60	14	23.33	44	12	27.27
Jardim das Oliveiras	250	77	30.8	222	54	24.32
Jardim Guanabara	78	20	25.64	249	51	20.48
Jardim Iracema	148	43	29.05	170	41	24.12
João XXIII	245	58	23.67	173	47	27.17
Joaquim Távora	145	39	26.9	93	21	22.58
Jóquei Clube	266	64	24.06	69	13	18.84
José Bonifácio	43	12	27.91	25	5	20
José de Alencar	142	35	24.65	18	4	22.22
Lagoa Redonda	287	67	23.34	64	11	17.19
Luciano Cavalcante	117	32	27.35	53	11	20.75
Manoel Sátiro	197	51	25.89	274	61	22.26
Manuel Dias Branco	17	6	35.29	15	5	33.33
Maraponga	77	19	24.68	63	16	25.4
Meireles	42	7	16.67	29	9	31.03
Messejana	867	239	27.57	157	39	24.84
Mondubim	423	110	26	356	88	24.72
Monte Castelo	175	60	34.29	32	4	12.5
Montese	294	95	32.31	77	26	33.77

Moura Brasil	25	9	36	8	2	25
Mucuripe	85	31	36.47	27	8	29.63
Olavo Oliveira	47	11	23.4	115	29	25.22
Padre Andrade	70	14	20	80	25	31.25
Palmeiras	764	196	25.65	159	47	29.56
Pan Americano	84	23	27.38	35	9	25.71
Papicu	120	29	24.17	31	5	16.13
Parangaba	244	60	24.59	121	31	25.62
Parque Araxá	89	20	22.47	35	10	28.57
Parque dois Irmãos	185	52	28.11	176	44	25
Parque Iracema	53	17	32.08	15	2	13.33
Parque Manibura	15	6	40	6	2	33.33
Parque Presidente Vargas	46	14	30.43	42	9	21.43
Parque Santa Maria	158	44	27.85	32	6	18.75
Parque Santa Rosa	81	17	20.99	75	16	21.33
Parque São José	93	20	21.51	90	17	18.89
Parquelândia	89	28	31.46	50	14	28
Parreão	80	24	30	20	3	15
Passaré	381	100	26.25	231	55	23.81
Paupina	157	44	28.03	101	28	27.72
Pedras	28	6	21.43	23	4	17.39
Pici	522	134	25.67	159	34	21.38
Pirambú	244	70	28.69	76	8	10.53
Planalto Ayrton Senna	171	37	21.64	137	28	20.44
Praia de Iracema	22	6	27.27	9	2	22.22
Praia do Futuro I	71	13	18.31	27	10	37.04
Praia do Futuro II	108	37	34.26	61	12	19.67
Prefeito José Walter	215	47	21.86	170	34	20
Presidente Kennedy	115	33	28.7	130	32	24.62
Quintino Cunha	124	38	30.65	225	53	23.56
Rodolfo Teófilo	252	61	24.21	89	17	19.1
Sabiaguaba	24	10	41.67	28	9	32.14
Salinas	18	6	33.33	5	1	20
São Bento	90	27	30	52	14	26.92
São Gerardo	59	16	27.12	32	6	18.75
São João do Tauape	233	61	26.18	132	28	21.21
Sapiranga Coité	160	52	32.5	63	19	30.16
Serrinha	284	76	26.76	129	29	22.48
Siqueira	149	50	33.56	216	50	23.15

Varjota	22	6	27.27	9	3	33.33
Vicente Pinzon	298	83	27.85	284	64	22.54
Vila Ellery	108	30	27.78	30	5	16.67
Vila Pery	185	49	26.49	71	16	22.54
Vila União	114	31	27.19	55	12	21.82
Vila Velha	126	31	24.6	541	131	24.21
